# Development of a factorial survey for use in an international study examining clinicians’ likelihood to support the decision to initiate invasive long-term ventilation for a child (the TechChild study)

**DOI:** 10.1186/s12874-022-01653-2

**Published:** 2022-07-21

**Authors:** Mary Brigid Quirke, Denise Alexander, Kate Masterson, Jo Greene, Cathal Walsh, Piet Leroy, Jay Berry, Lee Polikoff, Maria Brenner

**Affiliations:** 1 School of Nursing, Midwifery & Health Systems, University College Dublin, Dublin 4, Ireland; 2 University of Limerick, Limerick, V94 T9PX Ireland; 3 Maastricht University Medical Centre, Maastricht, Netherlands; 4 Boston Children’s Hospital, Boston, USA; 5 Brown University, Providence, USA

**Keywords:** Factorial survey, Pretesting, Survey design, Validity, Long-term ventilation, Technology dependence, Paediatric complex care, Chronically critically ill child

## Abstract

**Background:**

The decision to initiate invasive long-term ventilation for a child with complex medical needs can be extremely challenging. TechChild is a research programme that aims to explore the liminal space between initial consideration of such technology dependence and the final decision. This paper presents a best practice example of the development of a unique use of the factorial survey method to identify the main influencing factors in this critical juncture in a child’s care.

**Methods:**

We developed a within-subjects design factorial survey. In phase 1 (design) we defined the survey goal (dependent variable, mode and sample). We defined and constructed the factors and factor levels (independent variables) using previous qualitative research and existing scientific literature. We further refined these factors based on expert feedback from expert clinicians and a statistician. In phase two (pretesting), we subjected the survey tool to several iterations (cognitive interviewing, face validity testing, statistical review, usability testing). In phase three (piloting) testing focused on feasibility testing with members of the target population (*n* = 18). Ethical approval was obtained from the then host institution’s Health Sciences Ethics Committee.

**Results:**

Initial refinement of factors was guided by literature and interviews with clinicians and grouped into four broad categories: Clinical, Child and Family, Organisational, and Professional characteristics. Extensive iterative consultations with clinical and statistical experts, including analysis of cognitive interviews, identified best practice in terms of appropriate: inclusion and order of clinical content; cognitive load and number of factors; as well as language used to suit an international audience. The pilot study confirmed feasibility of the survey. The final survey comprised a 43-item online tool including two age-based sets of clinical vignettes, eight of which were randomly presented to each participant from a total vignette population of 480.

**Conclusions:**

This paper clearly explains the processes involved in the development of a factorial survey for the online environment that is internationally appropriate, relevant, and useful to research an increasingly important subject in modern healthcare. This paper provides a framework for researchers to apply a factorial survey approach in wider health research, making this underutilised approach more accessible to a wider audience.

**Supplementary Information:**

The online version contains supplementary material available at 10.1186/s12874-022-01653-2.

## Introduction

In recent decades, Paediatric Intensive Care Unit (PICU) mortality rates have decreased [1–3]. Concurrently, children with increasingly complex medical conditions are surviving and accordingly, post-PICU morbidity rates have increased [4, 5]. This has prompted a growing focus on bioethical discussions around issues such as survivability thresholds, quality of life, autonomy and other ways that the decision to initiate life sustaining technologies (such as invasive long-term ventilation (ILTV)) impact the child, their families and healthcare professionals [6, 7].

One of the most challenging issues in PICU care remains the issue of ILTV in children living with a range of complex medical needs. LTV is one of the most well-established forms of life sustaining medical technology dependence and dominates the research literature in this area [8]. While there has been an increase in children receiving non-invasive long-term ventilation (NI-LTV) the number of children initiated on ILTV has either remained static or decreased [9, 10]. Situations wherein ILTV is considered for a child living with complex medical needs are frequently the most medically challenging cases for clinicians to navigate with the child and family. The decision to initiate (or not initiate) ILTV can be an extremely challenging one for all involved [6, 11].

While the evidence-base quantifying the extent of increases in medical technology dependence has expanded, few studies have examined the liminal space between beginning to consider the initiation of technology dependence (such as ILTV) and the final decision being agreed upon [12, 13]. Findings from studies examining family and child participation in such decisions have highlighted that communication barriers, issues of trust, and a perceived lack of transparency create additional challenges for families during this difficult time [11]. Given the context of dynamic advances in medical technology, the potential for moral distress in clinicians is also an area of research coming to the fore [14].

In TechChild, we addressed the critical issues arising from the application of advances in life sustaining technology in paediatric medicine. This research programme increases insight into what influences the decision to initiate long-term technology dependence to sustain a child’s life and will develop a theory to explain the initiation of technology dependence in the context of diverse health, legal, and socio-political systems. The initial phase of TechChild involved interviews with clinicians (e.g., doctors, nurses, other MDTs, bioethicists) (*n* = 78) across several international hospital sites. This in-depth phenomenological investigation explored the experiences of clinicians with these children and their families during this decision-making period [6]. In the second phase of TechChild (the focus of the current paper), the investigation has shifted to examine the main influences on the decision to support (or not) the initiation of ILTV. Whilst rarely used in the clinical environment, the factorial survey technique is an exciting approach that has the capacity to address the goal of this phase of the project. Alongside providing a method that gives a wide reach in terms of sample, this approach adequately considers the complex and nuanced factors involved in the decision-making process. The factorial survey set out in this paper is unique in that it was conducted internationally and is the first that we are aware of to be undertaken on a critical care topic in paediatrics.

## Method

The primary aim of this article is to provide a narrative summary of the preparatory work undertaken to enable this next phase of TechChild. The secondary aims are to outline as a best practice example, our approach in the (1) design, (2) pretesting and (3) piloting of a factorial survey to identify the main factors that influence the decision to support initiation of ILTV. We detailed the process of stakeholder-informed refinement of survey content and the steps taken to ensure both validity and functionality in the current online environment, which is an adaptation of existing literature on this topic.

### Rationale for selecting a factorial survey-based approach

**Fig. 1:**
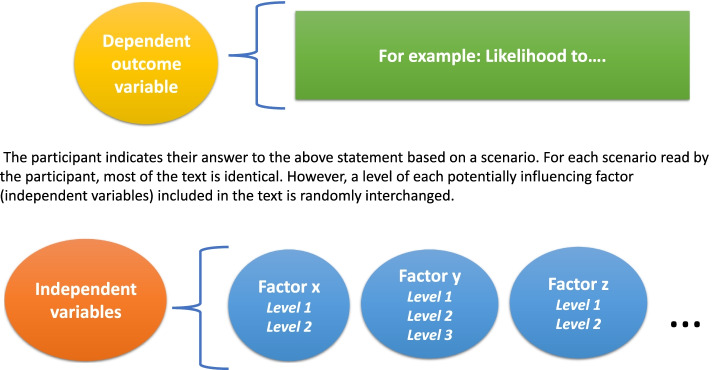
Overview of basic components of a factorial survey

The factorial survey approach is well suited as a tool for interrogation of the factors that determine the clinical decision to support (or not support) the initiation of technology dependence. It allows for random yet systematic manipulation of survey content such that the data collected from each participant becomes individually enriched and the risk of unobserved heterogeneity is reduced, and collinearity is minimised [15]. To briefly summarise its core structure, the factorial survey is a type of experimental vignette-based methodology [16] which takes the form of an ‘experiment within a survey’ [17, 18]. By identifying factors within a parameterised, controlled vignette, interchangeable levels of each factor can be randomly introduced that allow the researcher to present many iterations of the core vignette, differentiated according to the random incorporation of factor levels. This is shown in Fig. 1. The within-subjects design used in this study allows multiple responses to be collected from each respondent and analysed in a more experimental manner than is the case with a standard survey [17, 19].

In developing the TechChild factorial survey, we followed the conventional phases of survey development: Phase one - design, Phase two - pretesting and Phase three – piloting [20, 21]. The work within these phases was further guided by factorial survey literature [22–24]. Phase 1 formally defined the survey goal and its cognate dependent variable, as well as the appropriate mode and sample. As per the requirements of the factorial survey-based approach the independent variables were then defined and constructed as factors and factor levels. These factors were subject to refinement based on expert feedback from clinicians working with ILTV, and from a statistician. Thereafter, the standardised vignette text was established, and a total vignette population constructed with determination of the number of vignette sets and vignettes per respondent required. All methods were performed in accordance with the relevant guidelines and, where relevant, these are referenced throughout the text.

**Fig. 2:**
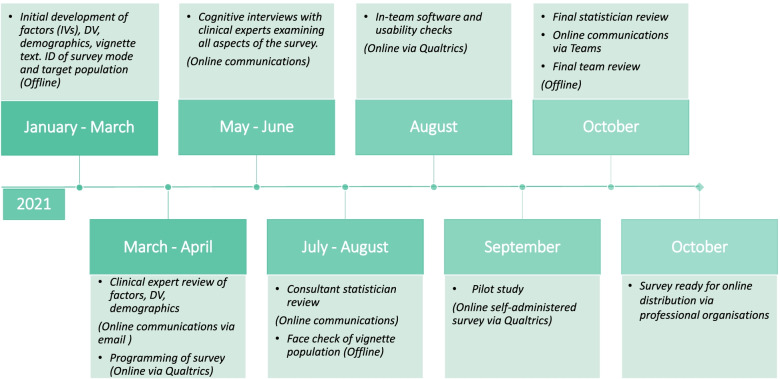
Factorial survey development in the TechChild research programme (2021)

On completion of phase one, the survey tool underwent several iterations before it was ready for use in data collection. Phases two and three were also guided by established survey pretesting checklists [25, 26] as well as additional checks identified in the factorial survey literature [22, 27, 28]. The study received approval from the then host institution’s Health Sciences Ethics Committee (Reference number: 190202). The content of the final survey was also reviewed by the data protection office oand deemed low risk. Figure 2 below illustrates these stages of development.

### Construction of the vignette population

Factors initially identified for inclusion were drawn from (1) available peer reviewed literature as well as (2) additional qualitative research [15, 23, 29]. Reviews of the literature (including published TechChild work [8, 12]) identified pertinent categories under which factors were grouped. Factors that emerged from the experiential interviews with clinicians (*n* = 78) in the first phase of the TechChild project (April 2020–November 2020) were also reviewed, and classified and refined alongside those identified from the literature. The rationale for each factor was identified in this two-pronged evidenced based way. This iterative process was a time and resource intensive exercise, which took 3 months (January–March 2021). The factors identified for potential inclusion in the survey were mapped out for presentation to the research team; and discussed at weekly team meetings. Additional specific team meetings were held at each stage of the vignette development.

Many experiences recalled by interviewees discussed the progression to invasive LTV (via tracheostomy) from NI-LTV. It was clear from the interviews with clinicians that there was often great complexity with respect to decision making regarding the transition from NI-LTV to ILTV. Hence many identifiable factors and factor levels for the survey were identified from these experiences. Accordingly, the scope of the factorial survey was narrowed to the initiation of ILTV via tracheostomy in children with complex medical needs. Both the interviews and the available literature pointed towards this type of scenario ranking highly as a source of difficulty in clinical decision-making regarding the initiation of technology dependence [4, 30, 31].

As outlined above, extensive discussion led to the identification of factors which had the potential to be included in the survey. The research team conducted a comprehensive literature and interview review relating to each factor to identify evidence-based rationales to support the inclusion of each one. The included factors were placed under one of the following categories: Child characteristics; Clinical characteristics; Family characteristics; Organisational characteristics. An initial draft of the survey was generated using this evidence-based work. Three age-based versions of the survey were generated to represent conditions across the lifespan of a child (infant; middle childhood and adolescent). The content of each survey comprised of (1) Vignettes (table of factors and vignette text) (2) A 10-point Likert response question (the dependent variable (DV)) (3) Participant demographics.

An initial table of factors and levels of factors were established for the three separate surveys. Also referred to as the vignette universe [32], the vignette population is the Cartesian product of the complete set of possible vignette permutations and the initial number for each survey is set out in Table 1.

**Table 1:** Initial IV (factors) population based on refinement of factor generation from literature and interviews

Survey	Number of factors	Levels of factors	Vignette population
**Infant (6–24 months)**	10	3x2x3x4x4x2x2x2x4x3	27,648
**Child and Adolescent (8–16 years)**	11	4x2x3x4x4x3x2x2x2x4x3	110,592
**Adolescent (13–17 years)**	11	3x2x3x4x4x3x2x2x2x4x3	82,944

### Pretesting content assessment

Stage one: Panel of clinical experts. Initial factors and level of factors were reviewed and critiqued by an international panel of three clinical experts who had previously engaged in the wider project as clinical consultants. All members of this expert panel had extensive clinical experience of working with children who require medical technology to sustain life. Factors were assessed for clarity, relevance, and appropriateness, with refinements made based on the panel’s combined feedback.

The clinicians were asked to comment on each factor (and level of factor) in terms of clarity, relevance, appropriateness, with an emphasis on face and external validity. Where a clinician suggested a factor should be modified or deleted, they made comments for the rationale and, where appropriate, provided alternative suggestions. Incorporating initial guidance from a statistical consultant, the research team examined the revised content. This review led to several broad revisions, summarised in Table 2.

**Table 2:** Overview of refinements required in the TechChild survey following review by panel of clinical experts

Refinement	Rationale
Number of surveys reduced to *n* = 2	For the diagnoses included, most initiation decisions would likely take place in (1) early childhood or (2) during adolescence.
Number of factors/levels of factors reduced	Some factor levels were amended to enhance their relevance.
Inclusion of more clinically relevant information in the scenario text	To provide more detail on the degree of care required.
Refinement of family circumstances/profile component	Many family circumstances were considered at early stages of survey development. However, it was noted that the meaning of these circumstances was often very context dependent. In addition, often such situations were considered an influence on post-initiation care rather than the decision to initiate.
Distance from tertiary centre changed to time from centre	Measures of distance represents very different meaning depending on the geographical context so travel time was considered a more valid measure.

Based on the feedback from the clinical experts, the content was reduced to two surveys and the levels of factors as well as the standard vignette text for both surveys were refined (Table 3):

**Table 3:** Summary of practitioner feedback-based factor refinement of each survey

Survey	Number of factors	Level of factors	Vignette population
Infant	8	3x2x2x3x2x2x3x3	1296
Adolescent	9	3x2x2x2x2x3x2x3x3	2592

Stage two: Cognitive interview-style assessments. Additional pretesting measures were considered essential to the validation of the survey. Cognitive interview-style survey assessments were completed with clinicians (*n* = 3) by a member of the research team. These consultative interviews were based on Tourangeau’s four-stage model of cognitive processing [33], further guided by established frameworks or guidelines [34, 35] and adapted to work with the nuances of a factorial survey. With regards to the latter point, the standard vignette content and repeated response question across all vignettes led to the team taking a more discursive vignette-by-vignette review, as opposed to a standard item-by-item examination observed in cognitive interviews of standard surveys. The interview protocol included observation checks, general questions to encourage think-aloud feedback and scripted yet flexible probes that were utilised where appropriate.

Two members of the previous expert panel as well as an additional clinical expert completed an interview. An important component of cognitive interviewing is the identification of comprehension differences and, given the international nature of the TechChild project, this was of particular importance to the team. Interviews were conducted remotely via the Zoom Meetings platform (San Jose, CA: Zoom Video Communications Inc.; https://zoom.us/) and the experts were sent instructions in advance. At the beginning of each interview, the interviewer explained how the review would proceed. The interviewer shared the screen and made notes as the interview progressed and the clinical expert considered each question. The interview was conducted as a consultation and no identifying or personal information were included in observations and notes. After three interviews, comments and suggestions were reviewed by the team and, where appropriate, the survey was amended.

Overall, the vignette format and content were reviewed favourably by all clinical experts for the survey development. In the infant survey, five factors remained unchanged, two factors underwent modification (rewording or removal of one level) and one factor was deleted. In the adolescent survey, five factors remained unchanged, three factors required minor modification and again one factor was deleted. It was agreed to remove the possibility of the diagnosis factor level ‘Rett Syndrome’ appearing alongside the factor ‘Adolescent’s expressed opinion’. The removal of this combination reduced the overall adolescent vignette population from 384 to 288 possible combinations.

In terms of the number of vignettes presented per participant, Sauer and colleagues [36] recommends limiting the number of vignettes per participant to less than 20 vignettes and no more than 11 factors to avoid cognitive overload, tiredness, boredom and/or inconsistent responses. Considering both the sensitive nature of the topic and number of factors included, eight vignettes (four from each age group) were presented during cognitive interviewing and this quantity was considered appropriate by the clinical expert interviewees. Based on the interview feedback, minor modifications were made to the vignette text to enhance flow, and the Likert scale options simplified (with the assent of the statistician) to enhance ease of response. Areas requiring amendment were categorised using Drennan’s cognitive interview field guide as a framework [37] (see Table 4).

**Table 4:** Overview of survey amendments required based on cognitive interview feedback (adapted from Drennan field guide [37])

Area of the interview	Interview feedback
Lexical problem/Comprehension	Minor lexical issues emerged that required clarification e.g. removal of abbreviations, minor wording changes, and clarification that parental dis/agreement referred to dis/agreement with the medical team.
	All clinical experts spent time reflecting on the term ‘complex medical needs’. Some alternatives were considered, such as chronically critically ill. However, all reviewers concluded that for the purposes of the survey, the existing term was most appropriate in the context of the question.
Logical problems	Layout of vignettes followed by questions on demographic profile was deemed to flow well. Some clinical experts felt the vignette text itself should have minor modifications to enhance flow as a case study presentation.
Retrieval from memory of relevant information /Cognitive load	The majority believed eight vignettes was appropriate. One indicated possible saturation at six (this was revisited at the pilot study stage and eight was considered feasible). The order of vignette presentation was amended
	All clinical experts suggested emphasising the need to instruct the survey participants to read each individual vignette carefully given the subtle differences when levels of factors were changed.
Clinically appropriate content	Some of the wording was changed to enhance clinical clarity
	All suggested the need to emphasise the chronic nature of deterioration
Additional areas (Cultural considerations; Response; Temporal issues, Comfort (i.e. did any aspects of the survey make the interview uncomfortable); inclusion/exclusion problems)	No issues emerged.

Stage three: Statistical review. CW reviewed the content and associated questions informed by feedback from the cognitive interviews. The two age-based surveys were retained; one factor (age) was reduced to two levels. A simplified 4-item Likert scale was also deemed most appropriate to encourage clearer decision-making by respondants [38]. No changes were made to the questions on the demographic profile of the participants. The changes were reviewed and confirmed by clinical experts and the researchers. A summary of the revised cartesian product of survey factors is set out in Table 5.

**Table 5:** Summary of cognitive interview-based factor refinement of each survey

Survey	Number of factors	Level of factors	Vignette population
Infant	7	2x2x2x2x2x3x2	192
Adolescent	8	2x2x2x2x2x2x3x2^a^	288

^a^ the survey was programmed so that the factor “Adolescent’s own opinion” did not appear in vignettes where the level “Rett Syndrome” appeared for the factor “Diagnosis”. This reduces the vignette population of the adolescent survey from 384 to 288

### Face validity

The entire vignette population for each of the surveys was generated using Python software (Python 3.9; Python Software Foundation, 2021). The vignettes were generated in this way to remove the randomising function of Qualtrics and to ensure that all vignettes were reviewed. Each vignette was assessed by two reviewers (a clinician and an academic) (*n* = 576). 

### Software, usability, and accessibility testing

The survey was set up and programmed using the Qualtrics platform (Qualtrics, Provo, UT). More detailed guiding information regarding this set up is set out in supplementary file 1.^1^ The survey was then assessed using the Qualtrics accessibility checklist and the team consulted with the Disability Office to confirm the survey passed accessibility standards. The survey content and format were amended where appropriate, for example the removal of a progress bar and the use of an accessibility-compliant font. Some accessibility improvements were not possible due to the nature of the survey design. For example, inclusion of a back button was not compatible with the randomiser function.

## Results

### Pilot study

An online international pilot study was completed in September 2021 with the purpose of assessing feasibility and identifying any possible issues that could negatively impact on data collection. The pilot was completed with a convenience purposive sample group who were members of the target population. All qualified clinical health professionals with experience of working with children at the time of the initiation of technology dependence were included. The survey (which included in a link with a PIL and informed consent form) was distributed via a gatekeeper, who worked as a nurse specialist in a large university hospital, to healthcare professional colleagues working in a PICU environment. The survey was also snowballed from this group (*n* = 18). As advised by the TechChild consultant statistician, data collection for the pilot study continued until sufficient data was gathered to assess the feasibility of the survey as well as appropriateness of the data format for analysis. This version of the survey included an optional open comment box after each item for any feedback and pilot participants were also invited to comment on the survey itself.

Of the 18 participants enrolled in the pilot study, 13 (72%) provided a response to at least seven of the eight vignettes. For 12 of the 18 participants (72%), a ≥ 90% vignette completion rate was obtained, suggesting no signal indicative of vignette saturation (i.e., the number of vignettes was appropriate). Feedback from the gatekeeper suggested that the most likely reason for an incomplete response was that the individual started the survey at work and was interrupted. The median length of time taken to complete the survey (those with a > 90% survey completion rate) was 10 minutes (mean = 18.2 minutes, SD = 17.6). This indicated that the instruction to participants that the survey would take approximately 10–15 minutes was accurate. Completion times did not indicate any specific issues. The demographic characteristics of participants who completed this section of the study (*n* = 12) are set out in Table 6.

**Table 6:** Demographic characteristics of in the pilot study

Characteristic (n)	
Age (12)	Mean = 41.5 years (SD = 11.1) Median = 39.5 years
Gender (12)	
Female	75% (9)
Male	17% (2)
Female and non-binary	8% (1)
Religious (12)	
Yes	33% (4)
No	54% (7)
Yes and not sure^a^	8% (1)
Discipline (12)	
Medical doctor	17% (2)
Registered nurse	42% (5)
Respiratory therapist/Physical therapist/Physiotherapist	25% (3)
Pharmacist	8% (1)
Dietician	8% (1)
Number of years in current position	Mean = 8.2 (SD = 4.3) Median = 6.5
Country currently employed (10)	
Australia	60% (6)
Ireland	20% (2)
USA	20% (2)

^a^In the pilot study response choices were not restricted to one option and one participant selected two options. This was amended to single choice in the final version

Seven of the 12 participants who completed the survey recorded comments. Only one participant commented on all of the vignettes. Thus, whilst a forced response option on the comments section may increase contextual information gathered on individual vignettes, our concern was that it may also adversely affect completion rates. If participants who chose not to comment were forced to contribute, given the open-ended nature of the comment question, they may choose to leave the study rather complete a section they did not want to answer.

Most comments focused on their response to a particular vignette rather than any issues with the survey, highlighting the value of including an option to add a comment box in the final survey. Some participants used the comments box to summarise the pertinent aspects of the vignette and others took the opportunity to explicitly set out their rationale for their response:

“Poor prognosis but family on board” (Respondent 9)

“I think you need to take the adolescent's opinion into account” (Respondent 4)

Where a need for additional information was indicated (*n* = 2), there were different opinions on what additional information might be useful. Further information on quality of life or social environment as well as additional clinical information were noted on individual vignettes:

“Need to explore how LTV will change quality of life, for the better or not” (Respondent 3)

“I think to fully decide on this I would want to have more information regarding the child’s development including physical function and cognitive function” (Respondent 9)

One respondent noted that after completing the demographic section, the importance of a family’s religious beliefs came to mind. Religious beliefs and many other important contributors were considered for inclusion by the research team. However, considering the complexities and nuances of these issues the decision to include the factor “Parental agreement/disagreement” was taken with this in mind because such individual family circumstances were often cited in the context of reasons for parental disagreement with the team.

Only two participants commented on the survey itself. These comments were minor issues with functionality (e.g., more than one option on some demographics questions could be selected) that were resolved. To ensure the pilot achieved the objectives, it was assessed based on existing pilot checklists [39].

The content of the final survey for distribution is set out in Table 7. This comprises the table of factors that were randomly interchanged for each participant in the main study along with the standard vignette text, response question and comment box following each vignette and the demographic questions.

**Table 7:** Content of final survey ready for distribution

Infant survey		Adolescent survey	
Factor	**Levels of factors**	**Factor**	**Level of factor**
Age (months)	**▪12** **▪24**	**Age (years)**	**▪13** **▪16**
Diagnosis	**▪Spinal Muscular Atrophy type 1** **▪Bronchopulmonary dysplasia**	**Diagnosis**	**▪Rett Syndrome** **▪Duchenne Muscular Dystrophy**
Prior BiPAP support (hours)	**▪12** **▪18**	**Prior BiPAP support (hours)**	**▪12** **▪18**
Parent coping	**▪Struggling to cope with care demands and had requested more home nursing hours** **▪Coping well with care demands**	**Parent coping**	**▪Struggling to cope with care demands and had requested more home nursing hours** **▪Coping well with care demands**
		**Adolescent’s own opinion**	**▪The adolescent has previously communicated that they want to have continuous invasive LTV via tracheostomy** **▪The adolescent has previously communicated that they do not want to have continuous invasive LTV via tracheostomy.**
Parent view (on decision to initiate)	**▪Agree** **▪Disagree**	**Parent view (on decision to initiate)**	**▪Agree** **▪Disagree**
Family network	**▪No** **▪A poor** **▪A good**	**Family network**	**▪No** **▪A poor** **▪A good**
Distance (from nearest tertiary care centre)	**▪Less than one hour** **▪More than three hours**	**Distance (from nearest tertiary care centre)**	**▪Less than one hour** **▪More than three hours**
Infant vignette text		**Adolescent vignette text**	
A [Age] month-old with a history of [Diagnosis] is currently ventilated in the PICU and is difficult to wean from the ventilator. This is the second time they have been ventilated in PICU since birth. This child has been on BiPAP [Prior BiPAP support] hours a day at home; there is an overall deterioration in their chronic respiratory condition. The degree of daily caregiving support for this child’s respiratory health increased in the month prior to admission, including increased nebulisation and suctioning. The family are receiving home care nursing hours, and, prior to the child’s current deterioration, the parents stated that they had been [Parent coping]. Consideration is now being given to continuous invasive long-term ventilation (LTV) via tracheostomy. The parents [Parent view] with the medical team on the need for initiating this treatment. The parents have [Family network] family network of support around them. This child and their parents live [Distance] from their nearest specialist care centre.		**A [Age] year-old with a history of [Diagnosis] is currently ventilated in the PICU and is difficult to wean from a ventilator. This is the second time they have been ventilated in the PICU in the last year. This adolescent has been on BiPAP [Prior BiPAP support] hours a day at home; there is an overall deterioration in their chronic respiratory condition. The degree of daily caregiving support for this adolescent’s respiratory health increased in the month prior to admission, including increased nebulisation and suctioning. The family are receiving home care nursing hours, and, prior to the adolescent’s current deterioration, the parents stated that they had been [Parent coping]** **Consideration is now being given to continuous invasive long-term ventilation (LTV) via tracheostomy. The parents [Parent view] with the medical team on the need for initiating this treatment. [Adolescent’s own opinion]. There is [Family network] family network of support around them. This adolescent and their parents live [Distance] from their nearest specialist care centre.**	
Response question (DV)			
Thinking about the above scenario, on a scale of 1–4, with 1 being extremely unlikely and 4 being extremely likely, how likely are you to support the initiation of continuous invasive long-term ventilation via tracheostomy for this child? 1. Extremely unlikely 2. Unlikely 3. Likely 4. Extremely Likely			
Please feel free to add comments regarding your choice of response			
Demographic section			
Your age		**(Years)**	
Gender		**Male; Female; Non-binary; Prefer to specify**	
Do you consider yourself as belonging to any particular religion or denomination?		**Yes, No, Not sure, Prefer to specify**	
What is your profession?		**Medical Doctor, Registered nurse, Nurse Practitioner, Physician Associate, Physical therapist, Physiotherapist, Respiratory therapist, Dietician, Pharmacist, Other (open box)**	
What is your job title?		**Response text box**	
No of years in current job position		**Drop down list of number options**	
No of years working with children with complex medical needs		**Drop down list of number options**	
Country currently employed		**Response text box**	
Through which professional organisation did you hear about this survey?		**Response text box**	
Any additional comments you would like to share		**Response text box**	

## Discussion

Traditional survey methods were not considered sufficient to identify the greatest influences on a clinician’s decision to support, or not support, the initiation of ILTV. In this methodological paper we adapted existing methodology for online use internationally with healthcare professionals who care for children at the time when ILTV initiation is being considered. Each stage of the factorial survey development and validation process has been set out, resulting in a field-ready tool that is feasible, appropriate, ethical and relevant.

This article contributes to the factorial survey literature by informing researchers of the practical steps involved when developing their own factorial survey in the healthcare area.

The development of, and pretesting approach to, a survey depends on the individual needs of the study. Whilst some aspects of a factorial survey are more complex (such as the interchangeable factors randomly presented to participants), other aspects of the design are easier to assess (for example the use of the same background vignette text and response question across vignettes). In the context of complex care medicine, the development and finalisation of factors/levels of factors was extremely time consuming compared to the other aspects of the survey development. In the current study, a great deal of consultation, discussion and subsequent refinement of the initial list of factors was required to produce a meaningful vignette population that is clinically relevant yet does not cognitively overburden the participant and lead to the use of heuristics [32, 36]. Each factor was considered both independently and relative to the other factors. The decision to include each level of factor and exclude others was a painstaking process, for example the ages and diagnoses chosen, consideration of novel therapies, and the family cultural/social characteristics.

Conversely, other aspects of pretesting were perhaps less burdensome than in other survey studies. There is debate in the literature on the appropriate sample size for cognitive interviews. Some studies suggest that similar numbers of participants to those in the pilot studies are the ideal; whilst other studies question this approach in terms of feasibility but also in terms of contribution [26, 39]. In reality, there is no consensus on optimal sample size; and critical appraisal by an experienced research team is required to determine an appropriate approach [37]. Similarly, the pilot study’s design, specific to a factorial survey, meant that informative analysis of small-scale data would be limited. Thus, the purpose of the pilot in our study was primarily to examine the feasibility and appropriateness of the survey, in addition to establishing that the suitability of the data format extracted from Qualtrics (Qualtrics, Provo, UT) would be suitable for the required analysis. This was particularly important to establish given the complex set up of the factorial survey design.

### Limitations and future directions for research

The factorial survey is a valuable tool in that it allows the flexibility to examine a multitude of factors in different ways. However, the nature of the factorial survey method also means that some formal tests of validity and reliability recommended in survey development, such as inter-rater reliability, test-retest and internal consistency, were not feasible; either because of the design or the sample or were inappropriate given the nature of the construct under examination.

This attribute of flexibility also means that the design features of a factorial survey across studies can differ substantially. This makes the approach of using a factorial survey sometimes challenging to appraise, compared to other factorial survey studies. It is particularly challenging to use by researchers who do not have a statistical or software background. The fast-paced development of survey administration tools such as Qualtrics (Qualtrics, Provo, UT) and RedCap (Research Electronic Data Capture) has limited the contribution of even relatively recent scientific papers on the factorial survey methodology, in terms of design, development and procedure. Some researchers have developed their own method of programming to address design limitations of standard software tools [40] a challenge for researchers who do not possess programming or software skills. Indeed, as alluded to in the paper, every stage of the development of the survey was time, skill and resource intensive for the TechChild team and thus this approach may not be a feasible method for researchers with less resources and support. Finally, this paper addresses many validity issues relevant to our current project but acknowledges that generalisability is limited to other factorial surveys that have similar design features.

## Conclusion

Developing a factorial survey for use in the paediatric critical care setting is novel. This paper explains the processes involved in the development of a factorial survey for the online environment that is appropriate, relevant, and useful on a subject which is becoming increasingly important in modern healthcare. This approach is potentially appropriate for use in other healthcare settings where decisions are made about sensitive issues. More in-depth information regarding the design, development and validity of different factorial survey designs are needed to support researchers in determining the needs of their study. The inclusion of more pretesting information in studies improves the ethical standards and design quality of a survey, thereby serving to protect participants, as well as increase confidence and trust in the research process.

## Supplementary Information

**Additional file 1.**

## Data Availability

The datasets generated and analysed during the current study are not publicly available due to the broader TechChild research programme is still in progress. Where possible this data will become available from the corresponding author on reasonable request.

## Abbreviations

ILTV: Invasive Long-Term Ventilation
NI-LTV: Non-invasive long-term ventilation
PICU: Paediatric Intensive Care Unit

## Footnotes

1 See Supplementary File 1
