# Supplementary material for: Development of a factorial survey for use in an international study examining clinicians’ likelihood to support the decision to initiate invasive long-term ventilation for a child (the TechChild study)
Source: BMC Med Res Methodol. 2022 Jul 21;22:198. doi: 10.1186/s12874-022-01653-2 (PMC9306171; doi:10.1186/s12874-022-01653-2)
Supplement: Supplementary file 1 — Additional file 1. [file 12874_2022_1653_MOESM1_ESM.docx]

**Supplementary file 1^1^**

**Qualtrics software set up and programming**

At the time of the study, the Qualtrics platform (Qualtrics, Provo, UT) was considered the most appropriate software for the needs of the study both in terms of functionality as well as organisation accessibility. The survey was set up and programmed on the Qualtrics platform using previous factorial surveys as a guide^1s,2s^ as well as support from the Qualtrics support team. Using the ‘randomizer’ function, each factor and level of factor was entered under ‘survey flow’ option in Qualtrics. This process was repeated forming eight blocks, one for each of the eight vignettes that would appear to each participant. Each participant received four infant vignettes and four adolescent vignettes and the blocks were set up so that every second vignette was from each group. The standardized background vignette text was also set up for all eight blocks and each factor level was programmed to randomly appear within the vignette text using the ‘piped text’ function on the Qualtrics system. To ensure a high level of orthogonality in factorial surveys, there has been a methodological shift towards a d-efficient design over the simple random allocation of factors^3s,4s^. However, the need for removal of illogical combinations in the current study created difficulties in using the d-efficient design. The intention to obtain a large sample as well as the inclusion of factors with varying numbers of levels all contributed to the decision to utilise random allocation in this study.

Software testing identified some minor issues through repeated testing of the survey and all were resolved. Where any illogical combinations were identified in consultation with the Qualtrics support team, these were programmed to not appear together using a ‘Then Branch If’ option under survey flow. Additional informal usability testing was completed by research team members and deemed appropriate. This was based on three considerations: (1) The simplicity of the survey layout from the perspective of the participant; (2) The limited size of the overall target population and (3) No usability issues were commented on during pretesting.

**1S.** McElhinney H, Taylor B, Sinclair M. Decision Making by Health and Social Care Professionals to Protect an Unborn Baby: Systematic Narrative Review. Child Care Pract. 2019;27:1-15

**2S.** Willis G. Questionnaire Pretesting. In Wolf, C., Joye, D., Smith, T., Fu, Y. The SAGE Handbook of Survey Methodology. 2016; 359-81. London: SAGE Publications Ltd

**3S.** Dülmer H. The Factorial Survey: Design Selection and its Impact on Reliability and Internal Validity. Sociol Methods Res. 2016;45(2):304-47

**4S.** Sauer C, Auspurg K, Hinz T, Liebig S, Schupp J. Methods Effects in Factorial Surveys: An Analysis of Respondents' Comments, Interviewers' Assessments, and Response Behavior. Berlin: German Socio-Economic Panel Study (SOEP); 2014. Contract No.: 629/2014.

|  |
| --- |
|  |
